# Reconstruction of Dorsal Wrist Defects

**Published:** 2015-09-07

**Authors:** Maelee Yang, Joseph Meyerson

**Affiliations:** The Ohio State University Wexner Medical Center, Columbus

**Keywords:** dorsal wrist, reconstruction, melanoma, flap coverage, skin grafts

**Figure:**
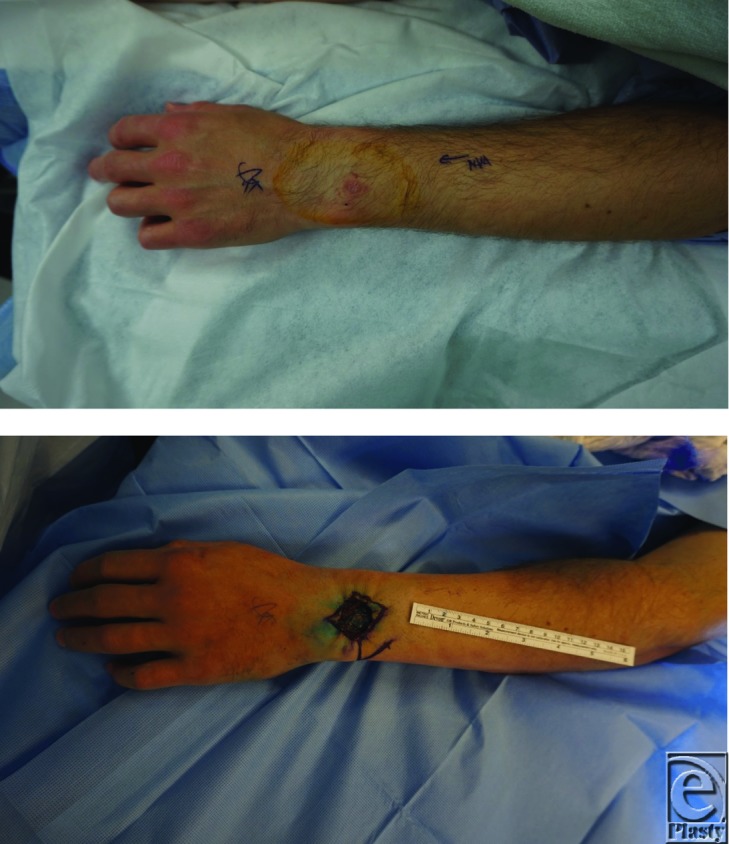


**Figure:**
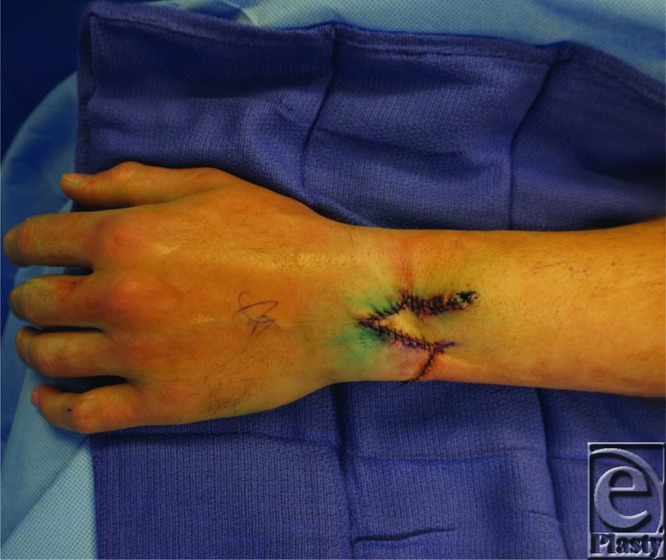


## DESCRIPTION

A 32-year-old man presented for excision of an irritated, growing mass on the left dorsal wrist.

## QUESTIONS

1. **What are the 3 most common skin malignancies of the hand and the wrist?**
2. **What are the challenges of dorsal wrist reconstruction?**
3. **What reconstructive options are used to repair dorsal wrist defects?**
4. **What are the potential complications of dorsal wrist reconstruction?**

## DISCUSSION

The differential diagnosis for the most common malignant skin lesions of the upper extremity includes, in order of incidence, squamous cell carcinoma, basal cell carcinoma, and melanoma. These cancers can typically be differentiated by their characteristic appearance and presentation; however, practitioners should be alerted to any atypical skin lesion. Although these are the most common malignancies seen on the dorsal arm, it is important to keep the differential broad, as the treatment can vary depending on the diagnosis. An excisional or incisional biopsy should be performed along with any indicated imaging studies for invasive tumors prior to any definitive reconstructive therapy.

The visible location and function of the dorsal wrist pose multiple challenges in reconstruction. The surrounding anatomy is responsible for both hand and forearm functions. Reconstructive efforts must preserve and protect underlying structures including bone, joints, muscles, tendons, nerves, and vasculature. The tenets integral to dorsal wrist reconstruction are that of durable and pliable coverage to withstand the constant use and movement of the wrist, repairing functional anatomy units, as well as correcting aesthetic challenges of matching skin texture, pliability, thickness, and coloring.

The reconstructive ladder should be considered when planning dorsal wrist reconstruction. Primary closure is the most desirable option, but for larger defects this is often not a feasible option. Healing by secondary intention is a viable option for smaller defects without any vital structure exposure. Skin grafting is a practical option when the defect does not involve bone or tendon lacking periosteum or peritenon, respectively. The surgeon should note that skin grafts may be susceptible to breakdown if the repair is in the dorsal wrist crease, an area of frequent motion and a location of watches and bracelets. Local and regional flaps are commonly the option of choice for dorsal wrist reconstruction. They can be used to cover vital structures, are more durable than grafts, have good color match, and can be sensate. Examples of these flaps include local cutaneous or perforator, posterior interosseous, radial forearm, ulnar forearm, and pronator quadratus flaps. Larger defects of the forearm may merit more extensive reconstruction using distant options such as the groin flap or free flaps.

It is important to keep potential complications in mind when considering each reconstructive route. Split-thickness skin grafts tend to contract and stiffen in comparison with full-thickness grafts, making them less suitable for dorsal wrist reconstruction. In addition, skin grafts can be less durable over time than other options. Distant flaps have subsequent donor sites that incur increased morbidity. As with all reconstructions, partial or complete flap loss may occur if tissue perfusion is poor. The plastic surgeon should ensure an optimized patient and wound bed prior to dorsal wrist reconstruction. In the case of malignancy, surgical margins should be negative and any traumatic wound should be thoroughly debrided to healthy tissue. Finally, scar direction and placement are important to consider as longitudinal scars that cross the dorsal wrist crease are prone to webbing.
